# Clinical characteristics and outcomes of diabetics hospitalized for COVID-19 infection: a single-centered, retrospective, observational study

**DOI:** 10.17179/excli2020-2988

**Published:** 2020-11-16

**Authors:** Asieh Mansour, Sayed Mahmoud Sajjadi-Jazi, Amir Kasaeian, Bardia Khosravi, Majid Sorouri, Fatemeh Azizi, Zeinab Rajabi, Fatemeh Motamedi, Azin Sirusbakht, Masoud Eslahi, Heila Mojtabbavi, Ali Reza Sima, Amir Reza Radmard, Mohhamad Reza Mohajeri-Tehrani, Mohammad Abdollahi

**Affiliations:** 1 Endocrinology and Metabolism Research Center, Endocrinology and Metabolism Clinical Sciences Institute, Tehran University of Medical Sciences, Tehran, Iran; 2 Cell Therapy and Regenerative Medicine Research Center, Endocrinology and Metabolism Molecular-Cellular Sciences Institute, Tehran University of Medical Sciences, Tehran, Iran; 3 Hematology, Oncology and Stem Cell Transplantation Research Center, Tehran University of Medical Sciences, Tehran, Iran; 4 Digestive Disease Research Center, Digestive Disease Research Institute, Tehran University of Medical Sciences, Tehran, Iran; 5 Department of Internal Medicine, Shariati Hospital, Tehran University of Medical Sciences, Tehran, Iran; 6 Radiology Department, Shariati Hospital, Tehran University of Medical Sciences, Tehran, Iran

**Keywords:** COVID-19, diabetes mellitus, DM

## Abstract

Some debates exist regarding the association of diabetes mellitus (DM) with COVID-19 infection severity and mortality. In this study, we aimed to describe and compare the clinical characteristics and outcomes of hospitalized COVID-19 patients with and without DM. In this single-centered, retrospective, observational study, we enrolled adult patients with COVID-19 who were admitted to the Shariati hospital, Tehran, Iran, from February 25, 2020, to April 21, 2020. The clinical and paraclinical information as well as the clinical outcomes of patients were collected from inpatient medical records. A total of 353 cases were included (mean age, 61.67 years; 57.51 % male), of whom 111 patients were diabetics (mean age, 63.66 years; 55.86 % male). In comparison to those without DM, diabetic patients with COVID-19 were more likely to have other comorbidities, elevated systolic blood pressure (SBP), elevated blood sugar (BS), lower estimated glomerular filtration rate (eGFR) and elevated blood urea nitrogen (BUN). The association of DM with severe outcomes of COVID-19 infection (i.e. mechanical ventilation, median length of hospital stay and mortality) remained non-significant before and after adjustments for several factors including age, sex, body mass index (BMI), smoking status, and comorbidities. Based on our results DM has not been associated with worse outcomes in hospitalized patients for COVID-19 infection.

## Introduction

Since December 2019, the new coronavirus named coronavirus disease 2019 (COVID-19) caused a public health emergency worldwide. With the outbreak of this virus in the world, the clinicians are increasingly confronted with problems and dilemmas associated with the possible effect of preexisting conditions such as hypertension, cardiovascular disease, cancer and diabetes mellitus (DM) on the course and outcome of COVID-19 infection (Zhu et al., 2020[18]). 

DM is a chronic disease with serious complications, affecting more than 463 million people around the world (Huang et al., 2020[7]). Whether DM is associated with severe and fetal COVID-19 infection remains controversial and population-based studies most from China have reported diverse findings; some show an increased mortality (Cao et al., 2020[2]) and severe COVID-19 infection among diabetic individuals (Wan et al., 2020[10]; Zhang et al., 2020[15]), whereas others find no association (Ruan et al., 2020[9]; Wang et al., 2020[11]).

The better understanding of factors related to the worse outcome in COVID-19 patients make clinicians able to select the vulnerable patients better and consequently improve medical care. Therefore, in the present study, we aimed to evaluate the clinical characteristics of hospitalized COVID-19 patients with and without DM and to investigate the association between DM and clinical outcomes among hospitalized COVID-19 patients.

## Methods

### Study design and participants

This single center, retrospective, observational study was performed at the Shariati hospital, Tehran, Iran. We retrospectively analyzed the data of adult patients (≥ 18 years old) with COVID-19 who were admitted to the hospital from February 25, 2020 (i.e. when the first patient was admitted), to April 21, 2020. Patients with possible COVID-19 infection were admitted to the hospital with the following criteria: 1) loss of consciousness, 2) respiratory rate > 24, 3) pulse rate > 90, 4) systolic blood pressure (SBP) < 90 mmHg, or 5) O_2_ saturation < 93 %. Chest computed tomography (CT) scan was performed for those patients with fever or respiratory symptoms. 

The diagnosis of COVID-19 was made based on the following criteria (Cariou et al., 2020[3]): 1) positive COVID-19 quantitative real-time reverse transcriptase-polymerase-chain-reaction (RT-PCR) test on samples from the nasopharynx or oropharynx or 2) clinical symptoms and chest CT findings (confirmed by two independent radiologist) indicative of COVID-19 infection. 

The study was approved by the ethics committee of Tehran University of Medical Sciences (code: IR.TUMS.VCR.REC.1399.002) and the requirement for written informed consent was waived by the ethics committee for this retrospective study.

### Data collection

The clinical, laboratory and radiologic data during hospital admission were reviewed and the following data were extracted from patients' medical records: 1) demographic data, 2) symptoms and signs at the time of hospital admission (i.e. fever, cough, dyspnea, sore throat, diarrhea, nausea/vomiting, anorexia, headache, weakness, myalgia, coryza, chills, chest pain, level of consciousness, blood pressure, pulse rate, respiratory rate and O_2_ saturation), 3) self-reported preexisting medical conditions (i.e. DM, hypertension, malignancy, kidney disease, ischemic heart disease [IHD], and cerebrovascular accident [CVA]), 4) laboratory test results within 24 hours of hospital admission (i.e. random blood sugar [BS], complete blood count with differential, coagulation profile, blood gas analysis, liver and renal function tests, electrolytes, and high-sensitivity C-reactive protein [hs-CRP], 5) chest CT images, and 6) clinical outcomes (i.e. length of hospital stay, mechanical ventilation, intensive care unit [ICU] admission, and death). All data were checked by two independent physicians.

### Definitions

DM was defined according to the self-reported history of DM or the use of antidiabetic medications (oral hypoglycemic agents or insulin). Those who had been smoking at least one cigarette per day at the time of evaluation were considered as current smoker. Immunodeficiency was defined based on the medical history of organ transplantation or using immunosuppressive drugs such as corticosteroids, methotrexate, azathioprine, cyclosporine, mycophenolate mofetil, tacrolimus, or sirolimus. Fever was defined by the oral temperature of 37.8 °C or higher. Shock was defined based on mean arterial blood pressure of less than 65 mmHg or SBP less than 90 mmHg (Brindley et al., 2006[1]). Both invasive and non-invasive mechanical ventilation were considered as mechanical ventilation treatment.

To calculate the CT severity score, each lung was divided into three zones, (1) upper zone (above the carina), (2) middle zone (from the carina to the inferior pulmonary vein) and (3) lower zone (below the inferior pulmonary vein). Then based on the severity of parenchymal involvement, the following scores were assigned for each zone: 0, no involvement; 1, 1-25 % involved; 2, 26-50 % involved; 3, 51-75 % involved; 4, 76-100 % involved. Finally, overall chest CT severity score was estimated by summing the scores from all six zones (range of possible score, 0-24).

### Statistical analyses

Continuous variables were presented as mean (standard deviation, SD) or median (interquartile range, IQR). Categorical variables were presented as number (%). Parametric and non-parametric tests including the independent t test, Mann-Whitney U test, χ² test, or Fisher's exact test were used to compare differences between variables where appropriate.

To examine whether DM is the risk factor for worse outcomes, multivariable logistic regression models were used. Adjusting covariates were selected based on the parameters associated with severe outcomes suggested by previous studies, including age, sex, body mass index (BMI), smoking status, and comorbidities (e.g. hypertension, IHD, CVA, malignancy, chronic kidney disease (CKD)/ dialysis, and immunodeficiency) (Zheng et al., 2020[16]). All tests were two-sided, and a *P* value less than 0.05 was defined as statistically significant. The Stata 11 software (StataCorp, Texas, USA) was used for statistical analyses.

## Results

The data of 353 patients (203 men and 150 women) hospitalized for COVID-19 infection were included (mean age, 61.67 years [range, 18-97 years]). The COVID-19 RT-PCR test was done for all patients and chest CT scan was performed in 239 patients. Of these 353 patients, the diagnosis of COVID-19 was based on positive RT-PCR in 164 patients and the remaining patients were diagnosed based on the clinical symptoms and chest CT scan indicative of COVID-19 infection. 

Based on past medical history, 111 (31.44 %) patients had DM and among them COVID-19 infection was diagnosed according to positive RT-PCR test in 52 cases. Other comorbidities including hypertension, IHD, CVA, malignancy, CKD/dialysis, and immunodeficiency were identified in 220 (62.32 %) cases. In addition to DM, hypertension (36.54 %) and IHD (25.78 %) are the most common comorbidities in hospitalized COVID-19 patients (Table 1(Tab. 1)). In comparison to non-diabetic patients, those with DM had a higher proportion of comorbidities (*P*<0.001), including IHD (*P*=0.006), hypertension (*P*<0.001), CKD/dialysis (*P*=0.002) and malignancy (*P*=0.035) (Table 1(Tab. 1)).

The most common symptoms at the time of admission were dyspnea (56.09 %), fever (50.42 %), cough (50.14 %), and weakness (41.08 %). With the exception of higher SBP among diabetic patients (*P*=0.017), neither of symptoms nor signs were significantly different between patients with DM than those without (Table 1(Tab. 1)).

Apparently, diabetic patients had higher levels of random BS (median 227.5 mg/dL in diabetics vs. 109 mg/dL in non-diabetics). In addition, renal function indices including estimated glomerular filtration rate (eGFR) and blood urea nitrogen (BUN) were significantly different in diabetic patients in comparison to those without DM; higher BUN (*P*=0.015) and lower eGFR (*P*=0.018) in diabetic patients (Table 2(Tab. 2)). 

In the course of hospital admission, 122 (34.56 %) patients receive ICU care and 111 (31.44 %) patients required mechanical ventilation. The median length of hospital stay was 4 days (IQR 2-9). Neither of outcomes including ICU admission, mechanical ventilation, median length of hospital stay and mortality was significantly different between patients with DM and those without (Table 1(Tab. 1)). The relationship between DM and clinical outcomes (mechanical ventilation, median length of hospital stay and mortality) remained non-significant after adjustments for several factors including age, sex, BMI, smoking status, and comorbidities (Table 3(Tab. 3)).

In patients with DM, mortality rates were higher among males (*P*=0.005). To compare clinical characteristics of survivors and non-survivors with DM, non-survivors were older (mean (SD), 68.93 (13.07) vs. 61.88 (13.01); *P*=0.005) and were more likely to have comorbidities (92.86 % vs. 69.88 %; *P*=0.014) especially immunodeficiency (21.43 % vs. 1.2 %; *P*=0.001). In addition, non-survivors were more frequently unconscious (35.71 % vs. 3.61 %; *P*<0.001) and had a lower SBP (median, 129 vs. 136 mmHg; *P*=0.031) than survivors at the time of hospital admission. Among the 111 patients who were diabetics, 38 (34.23 %) patients were treated in the ICU and 35 (31.53 %) patients received mechanical ventilation. Compared with survivors, non-survivors were more likely to admit to the ICU (100 % vs. 12.05 %; *P*<0.001), and received mechanical ventilation (100 % vs. 8.43 %; *P*<0.001) (Table 4(Tab. 4)). As shown in Table 5(Tab. 5), several laboratory tests were significantly different between diabetic survivors and non-survivors. The neutrophil/lymphocyte ratio (*P*=0.022), creatinine (*P*=0.002), and BUN (*P*=0.004) were significantly higher in diabetic non-survivors. Furthermore, platelet count (*P*=0.014), pCO_2 _(*P*=0.039), bicarbonate (*P*=0.039) and eGFR (*P*=0.004) were significantly lower in diabetic non-survivors (Table 5(Tab. 5)). 

See also the Supplementary data.

## Discussion

In this report, we describe the baseline clinical features, laboratory parameters and the main outcomes of 353 patients with or without DM, who were hospitalized with the diagnosis of COVID-19 in Shariati hospital. Our study indicated that DM is not associated with the main adverse clinical outcomes, including higher length of hospital stay, need to mechanical ventilation and mortality in hospitalized COVID-19 patients before and after adjustments for several factors.

COVID-19 is a novel disease and our knowledge about the possible risk factors related to disease severity and death are limited. Several studies evaluated the association between DM and COVID-19 severity and mortality with inconsistent results (Cao et al., 2020[2]; Du et al., 2020[5]; Huang et al., 2020[7]; Zhang et al., 2020[14]). Some of the studies indicated that DM was associated with significant increase in composite adverse clinical outcomes and death in COVID-19 patients. For example, in a study conducted by Zhou et al. 31 % of those dying with COVID-19 infection were diabetic (*P*=0.0051) (Zhou et al., 2020[17]). In another study, Cao et al. showed a higher prevalence of DM in COVID-19 patients who died (5.9 % in survivors vs. 35.3 % in non-survivors; *P*=<0.001) (Cao et al., 2020[2]). Yan et al. also indicated higher mortality rate among hospitalized COVID-19 patients with DM (Yan et al., 2020[13]). Furthermore, in a recent published meta-analysis including 6452 COVID-19 patients from 30 observational retrospective studies, the underlying diabetic disease was associated with severe COVID-19 infection (RR, 2.45 95 % CI: 1.79-3.35; *P*<0.001), acute respiratory di-stress syndrome (ARDS) (RR, 4.64 95 % CI: 1.86-11.58; *P*=0.001) and higher mortality (RR, 2.12 95 % CI: 1.44-3.11; *P*<0.001) (Huang et al., 2020[7]). The following mechanisms are suggested by different studies through which DM plays a role in COVID-19 severity and mortality: 1) compromising the immune response (Huang et al., 2020[7]), 2) reduction of pulmonary function (Yan et al., 2020[13]), 3) induction of hypercoagulability states (Guo et al., 2020[6]). 4) induction of an inflammatory state with increased production of inflammatory markers including interleukin (IL)-6, IL-1 and tumor necrosis factor-alpha (TNF-α) (Huang et al., 2020[7]), and 5) its association with micro- and macrovascular complications and other comorbidities (Cariou et al., 2020[3]).

In contrast, the results of other studies were in line with the present study, indicating that DM is not a risk factor for the disease severity and mortality among COVID-19 patients. For instance, Zhang et al. showed that DM was not associated with disease severity in COVID-19 patients (Zhang et al., 2020[14]). Chen et al. also found no association between DM and COVID-19 death in 274 hospitalized patients (Chen et al., 2020[4]). Moreover, in a prospective study conducted by Du et al., DM was not associated with higher mortality rate (Du et al., 2020[5]). 

Some of these controversies regarding the association of DM and COVID-19 outcome may arise from differences in baseline characteristics of participants, DM definition criteria, and the criteria used for diagnosis of COVID-19 infection in different studies (Cariou et al., 2020[3]; Guo et al., 2020[6]; Yan et al., 2020[13]). In addition, as shown by our report, diabetic patients with COVID-19 were more likely to have other comorbidities including HTN, IHD, malignancy, and CKD than those without, suggesting that some of the reported worse outcome in diabetic patients may be related to other comorbidities they usually had. However, most of those reports that conclude DM as a risk factor for disease severity and mortality in COVID-19 patients do not adjust the results to other parameters (i.e. age, sex, other comorbidities, and drugs) to determine the net effect of DM on clinical outcomes (Cao et al., 2020[2]; Roncon et al., 2020[8]; Wang et al., 2020[12]; Zhang et al., 2020[15]; Zhou et al., 2020[17]).

As shown by Huang et al., the association between DM and poor outcome among COVID-19 patients may be modified by other factors such as age and hypertension. They demonstrated that DM had less effect on COVID-19 severity in older and hypertensive patients. In other words, the effect size of DM is higher in younger patients (< 55 years) and those without hypertension (Huang et al., 2020[7]). The lack of association between DM and poor clinical outcomes in our study may also be explained by this fact that most of our COVID-19 patients including diabetic patients were old (76.58 % of diabetic patients were ≥ 55 years) and hypertensive (57.66 % of diabetic patients).

Compatible with previous findings, our results indicated that the mortality rate was higher among older male patients (Cariou et al., 2020[3]; Yan et al., 2020[13]). In addition, based on our results, diabetic patients who were smokers and those with other comorbidities especially immunodeficiency had higher mortality rate. Loss of consciousness and lower SBP at the time of hospital admission were also related to poor clinical outcome in diabetic COVID-19 patients. No agreement was present among various studies regarding the best laboratory tests which predicted outcomes of COVID-19 in diabetic patients at the time of hospital admission (Cariou et al., 2020[3]; Yan et al., 2020[13]). However, our results showed that lower platelet count, pCO_2_ and bicarbonate as well as higher neutrophil/lymphocyte ratio, BUN and creatinine at the time of hospital admission were associated with higher mortality in diabetic patients.

The current study had several limitations. First, the small sample size. Second, the retrospective nature of the study. Third, the definition of DM in our study was made based on self-reported medical history (lack of hemoglobin A1c measurements in all patients), so some of diabetic patients may be missed. Fourth, the inclusion of those with either positive COVID-19 RT-PCR test or clinical symptoms and chest CT findings indicative of COVID-19 in our study. Nevertheless, in our study, by repetition of the analyses only in those patients with positive COVID-19 RT-PCR test (164 patients), the association between DM and clinical outcomes were not changed (data not shown).

In summary, our findings suggest that DM is not associated with poor clinical outcomes among hospitalized patients with COVID-19. However, due to limitations mentioned above, our findings should be interpreted cautiously and further well-designed prospective studies are needed to confirm the data.

## Notes

Asieh Mansour and Sayed Mahmoud Sajjadi-Jazi contributed equally as first authors.

## Ethics approval and consent to participate

The study was approved by the ethics committee of Tehran University of Medical Sciences (code: IR.TUMS.VCR.REC.1399.002) and the requirement for written informed consent was waived by the ethics committee for this retrospective study.

## Consent for publication

Not applicable

## Competing interests

None of the authors have any conflicts of interest or financial ties to disclose.

## Funding

None

## Authors' contributions

A.M. and S.M.S.J. study conception and design, analysis and interpretation of data, drafting of manuscript, critical revision. A.K. analysis and interpretation of data. B.K., F.A., Z.R., F.M, A.S., M.E, and H.M. acquisition of data. M.S. study conception and design, analysis and interpretation of data. A.R.S., A.R.R., and M.R.M.T. critical revision. M.A. study conception and design, analysis and interpretation of data, critical revision.

## Supplementary Material

Supplementary data

## Figures and Tables

**Table 1:**
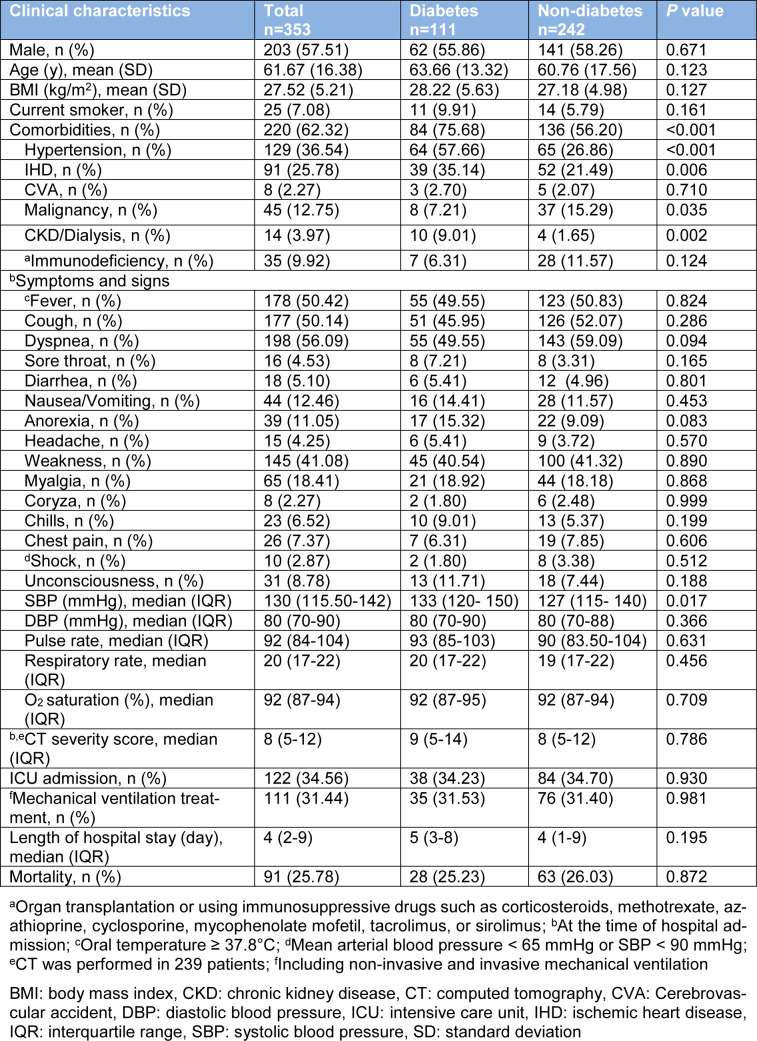
Clinical characteristics and outcome of hospitalized COVID-19 patients based on the presence of diabetes

**Table 2:**
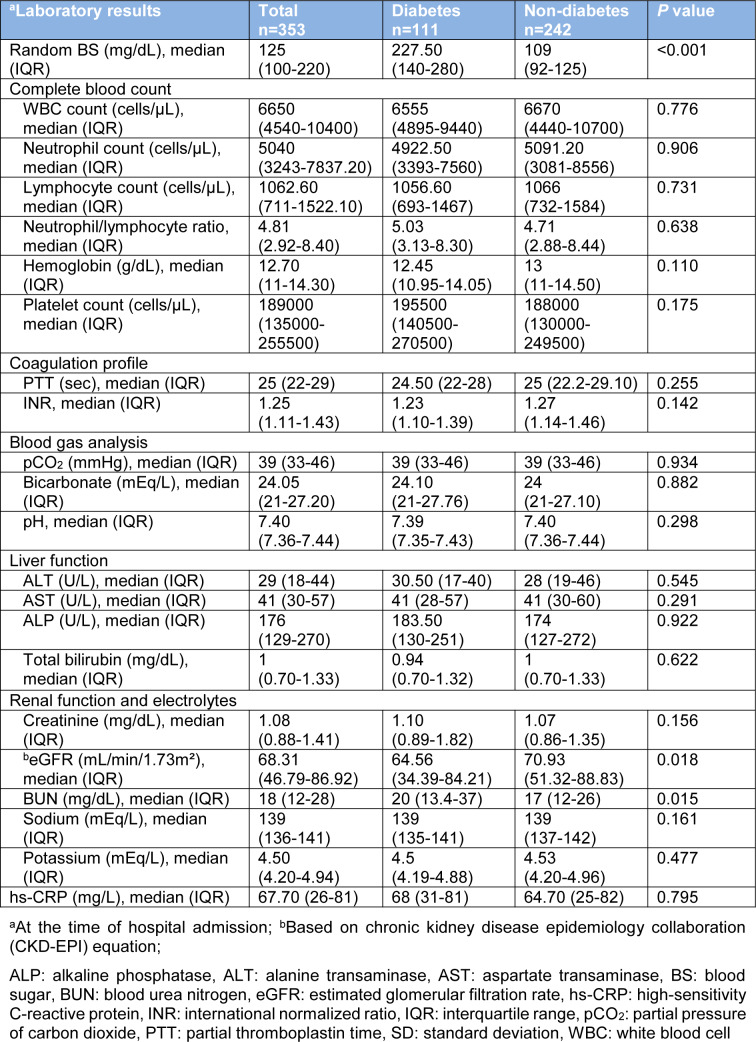
Laboratory results among hospitalized COVID-19 patients with or without diabetes

**Table 3:**
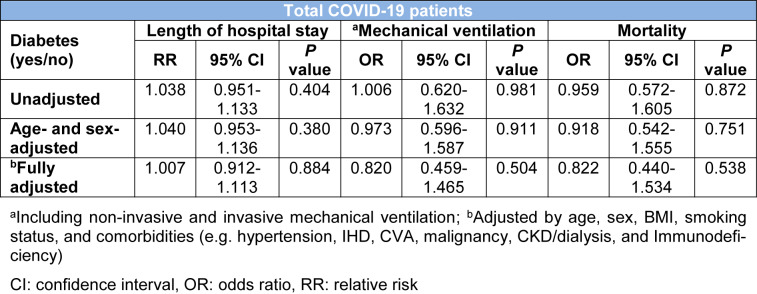
Assessing the association of diabetes with length of hospital stay, mechanical ventilation, and death in hospitalized patients with COVID-19 using the multivariable logistic regression analysis

**Table 4:**
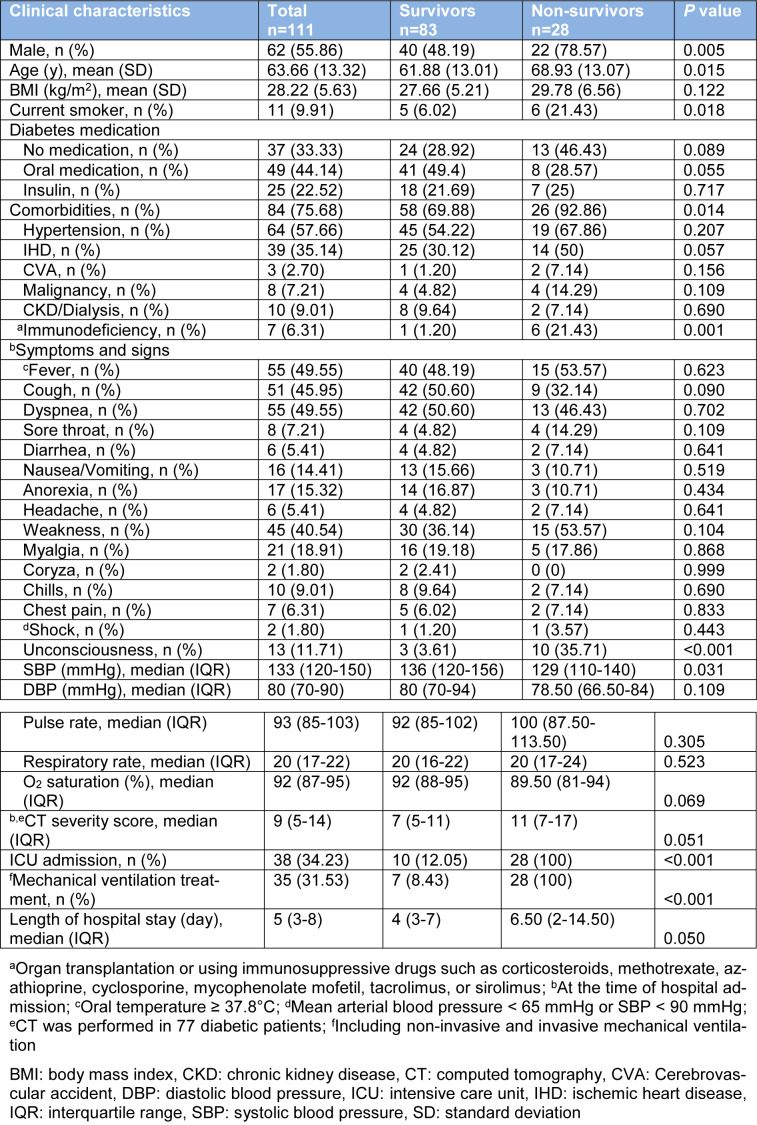
Clinical characteristics of hospitalized diabetic COVID-19 patients categorized based on mortality outcome

**Table 5:**
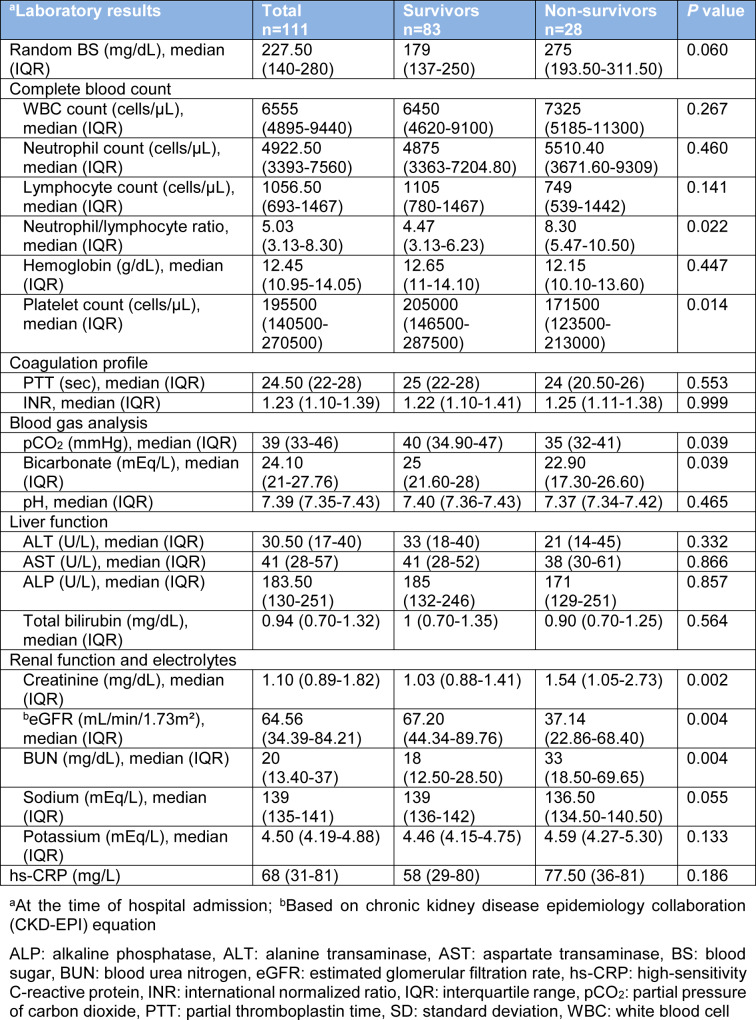
Laboratory results among hospitalized diabetic COVID-19 patients categorized based on mortality outcome
